# Placebo induced expectations of mood enhancement generate a positivity effect in emotional processing

**DOI:** 10.1038/s41598-022-09342-2

**Published:** 2022-03-29

**Authors:** Joshua Baker, Matthias Gamer, Jonas Rauh, Stefanie Brassen

**Affiliations:** 1 Department for Systems Neuroscience, University Hospital Hamburg-Eppendorf, Hamburg, Germany; 2 Department of Psychology, University of Würzburg, Würzburg, Germany

**Keywords:** Neuroscience, Psychology

## Abstract

A perceptual bias towards negative emotions is a consistent finding in mood disorders and a major target of therapeutic interventions. Placebo responses in antidepressant treatment are substantial, but it is unclear whether and how underlying expectancy effects can modulate response biases to emotional inputs. In a first attempt to approach this question, we investigated how placebo induced expectation can shape the perception of specific emotional stimuli in healthy individuals. In a controlled cross-over design, positive treatment expectations were induced by verbal instructions and a hidden training manipulation combined with an alleged oxytocin nasal spray before participants performed an emotion classification task on happy and fearful facial expressions with varying intensity. Analyses of response criterion and discrimination ability as derived from emotion-specific psychometric functions demonstrate that expectation specifically lowered participants’ threshold for identifying happy emotions in general, while they became less sensitive to subtle differences in emotional expressions. These indications of a positivity bias were directly correlated with participants’ treatment expectations as well as subjective experiences of treatment effects and went along with a significant mood enhancement. Our findings show that expectations can induce a perceptual positivity effect in healthy individuals which is probably modulated by top-down emotion regulation and which may be able to improve mood state. Clinical implications of these promising results now need to be explored in studies of expectation manipulation in patients with mood disorders.

## Introduction

While there is a large body of research addressing the impact of expectation on pain perception^1–4^, far less is known about the nature of expectation effects on mood states and emotional processing. Up to 70–80% of the benefits of antidepressant treatments have been attributed to placebo responses^5–7^, indicating the urgency of minimizing placebo responses in clinical trials but maximizing these responses in clinical practice^8^. A so called negativity bias in attention, i.e., increased attention towards negative information and decreased attention towards positive information, is a key feature in clinical depression^9^ and a critical modulator in the maintenance and prognosis of depressive symptoms^10,11^. For instance, compared to healthy adults, individuals in a negative mood state are often slower at identifying positive emotions^12^, focus more strongly on sad facial expressions^13^, are more sensitive to negative facial expressions^14,15^, and interpret neutral facial expressions more often as negative^16^. Antidepressant treatment has been shown to decrease such a bias^17,18^ but the role of expectation in this improvement is unknown. Promising findings in previous studies indicate that placebo effects in emotional processing activate top-down regulation on the down-stream processing of sensory input^19–22^. Such a regulatory process is also thought to be involved in the generation of a positivity effect, i.e. an enhanced processing of positive compared to negative information^23,24^) which in turn has been associated with better mood state^22,25^. Thus, one could speculate that expectation alone can activate a preferential processing of specific emotional information that may promote mood enhancement.

In the context of placebo analgesia, it has been shown that the induction of treatment expectation is more effective when combining false information about treatment efficacy with an active manipulation than just informing participants verbally^26^. Such an active manipulation through conditioning and learning procedures is well established for testing placebo analgesia^27–29^ but seems to be more complicated to apply in the affective system. The few existing studies simulating antidepressant or mood-enhancing effects within an experimental setup^22,30^ suggest that placebo effects in the affective domain may similarly depend on such learning effects.

In the current study, we aimed to investigate whether the induction of positive expectation can generate a positivity effect in healthy individuals. Specifically, within a controlled, cross-over design, positive expectation was induced by combining an alleged intra-nasal placebo “oxytocin” treatment with an active (hidden) manipulation of a training set that facilitated the detection of positive stimuli. After placebo induction or a control condition, participants performed an emotion classification task in which morphed faces with varying degrees of fearful or happy facial expressions had to be classified. We hypothesized that placebo expectation would increase participants’ tendency to classify even subtle expressions as being happy while fearful expressions become less salient. We expected that such an induced positivity effect would be directly related to reported treatment expectations and mood changes. By regressing classification accuracies of the placebo on the control conditions, we were able to generate emotion-specific psychometric response functions that allowed us to disentangle expectation effects on response tendencies (the intercept) and discrimination ability (the slope). The latter is particularly interesting given controversial evidence about whether expectation can actually improve cognitive functions^31,32^.

## Results

### Placebo induction enhances positive expectation, mood state and post-treatment experience

**Figure 1:**
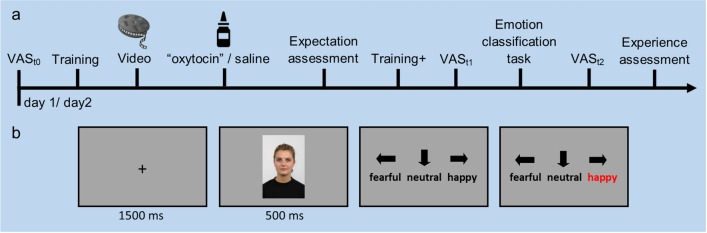
Study design and paradigm. (**a**) Procedure timeline. (**b**) Timing of the emotion classification paradigm. VAS = visual analogue scale. Face depicts an example stimulus used with permission from the Radboud Face Database.

Forty healthy young adults (age: 25.6 ± 4.4 years; 21 women) participated in this study. All participants underwent a randomized, controlled cross-over design with two study days on which they performed an emotion classification task as well as expectation and mood assessments. On one of these days, participants received an alleged “oxytocin” intranasal treatment combined with an active simulation of treatment effects (training + , see  “Methods” section for details). On the control day, participants were told to receive a pure saline nasal spray (Fig. 1).

**Figure 2:**
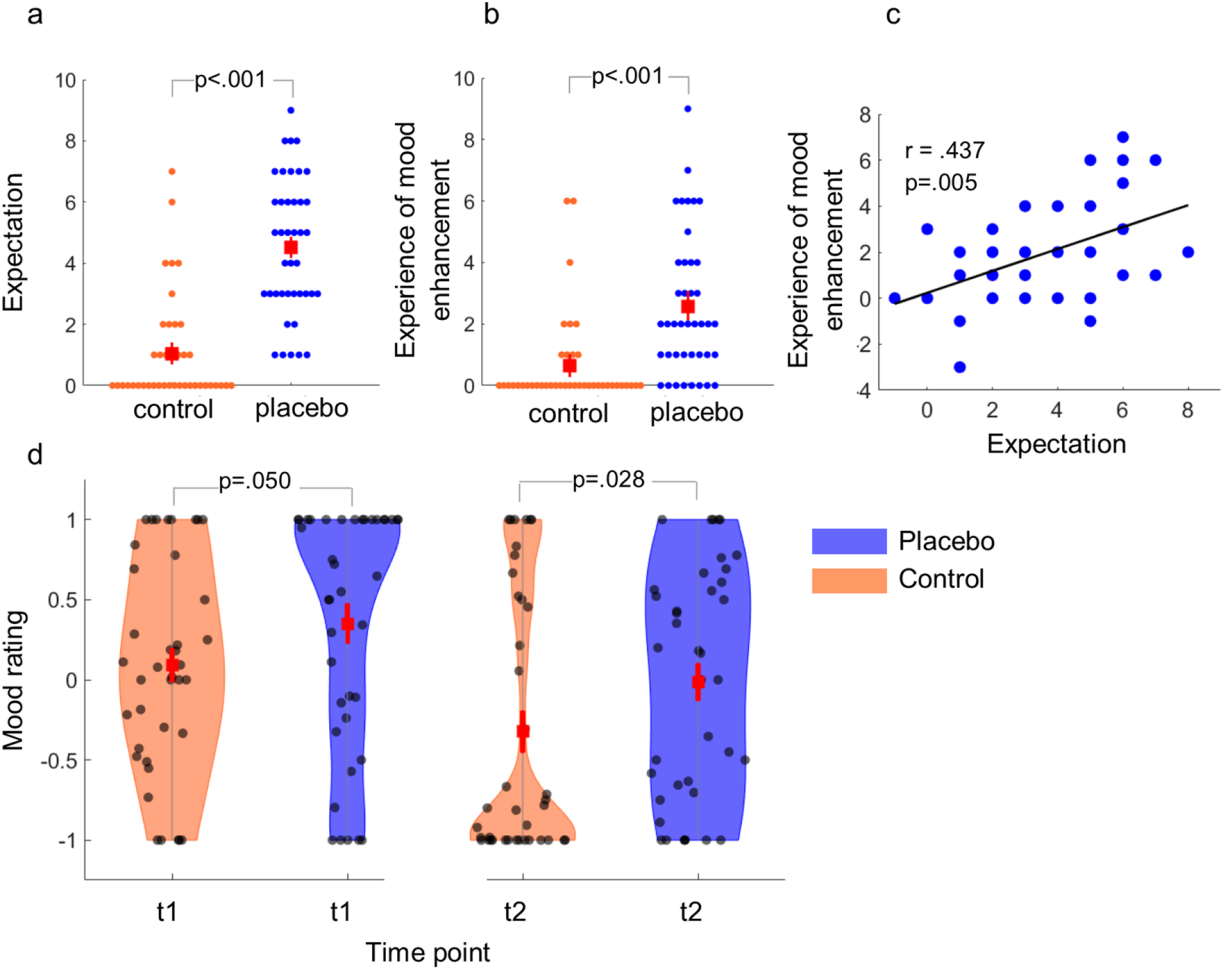
Expectation, experience, and mood ratings. (**a**,**b**) Positive treatment expectation and experience ratings. Both expectation and experience ratings increased in the placebo (blue) condition relative to the control (orange) condition. (**c**) Correlations between differences in expectation and experience ratings. Higher expectations of a positive mood change predicted higher experience of a positive mood change. (**d**) VAS mood ratings. Participants demonstrated a higher mood rating in the placebo condition relative to the control condition at both time points. Data are range normalized and baseline corrected. Mean (red square) and s.e.m. are shown in the center of each subplot.

Expectation ratings of positive treatment effects (i.e., improvement in mood) were significantly higher in the placebo compared to the control session (t(39) = 10.17, p < 0.001, Fig. 2a) indicating a successful expectation induction. Baseline mood state was assessed via VAS ratings before treatment application and did not differ between both study days (t(39) = 0.864, p = 0.393). Follow-up mood assessments before (t1) and after (t2) the emotion classification task demonstrate a strong session effect. Specifically, rmANOVA on baseline and range corrected VAS values with the factors condition (control/placebo) and time (t1/t2) revealed a significant condition effect (F[1, 37]  = 5.66, p = 0.023, η^2^_p_ = 0.133) that was due to a stronger mood enhancement on the placebo [M = 0.192, SD = 0.68] compared to the control session [M = −0.144, SD = 0.68]. This effect was significant at t1 and t2 (Fig. 2d). In addition, we observed a significant main effect of time (F[1, 37] = 32.83, p < 0.001, η^2^_p_ = 0.470) with higher mood ratings at the first [M = 0.22, SD = 0.49] relative to the second time point [M = −0.17, SD = 0.60], possibly indicating habituation effects. Since recapitulated treatment experience is an important prior for future treatment effects, we also assessed participants overall treatment experience at the end of each session. Here, participants reported a stronger experience of a positive mood increase at the end of the placebo session [M = 2.47, SD = 2.25] as compared to the control session [M = 0.62, SD = 1.49] (t(39) = 5.28, p < 0.001, Fig. 2b). This effect was directly correlated with the increase of positive expectation as assessed after treatment application (r(40) = 0.437, p = 0.005, Fig. 2c).

### Positive expectation facilitates the identification of happy facial emotions

**Figure 3:**
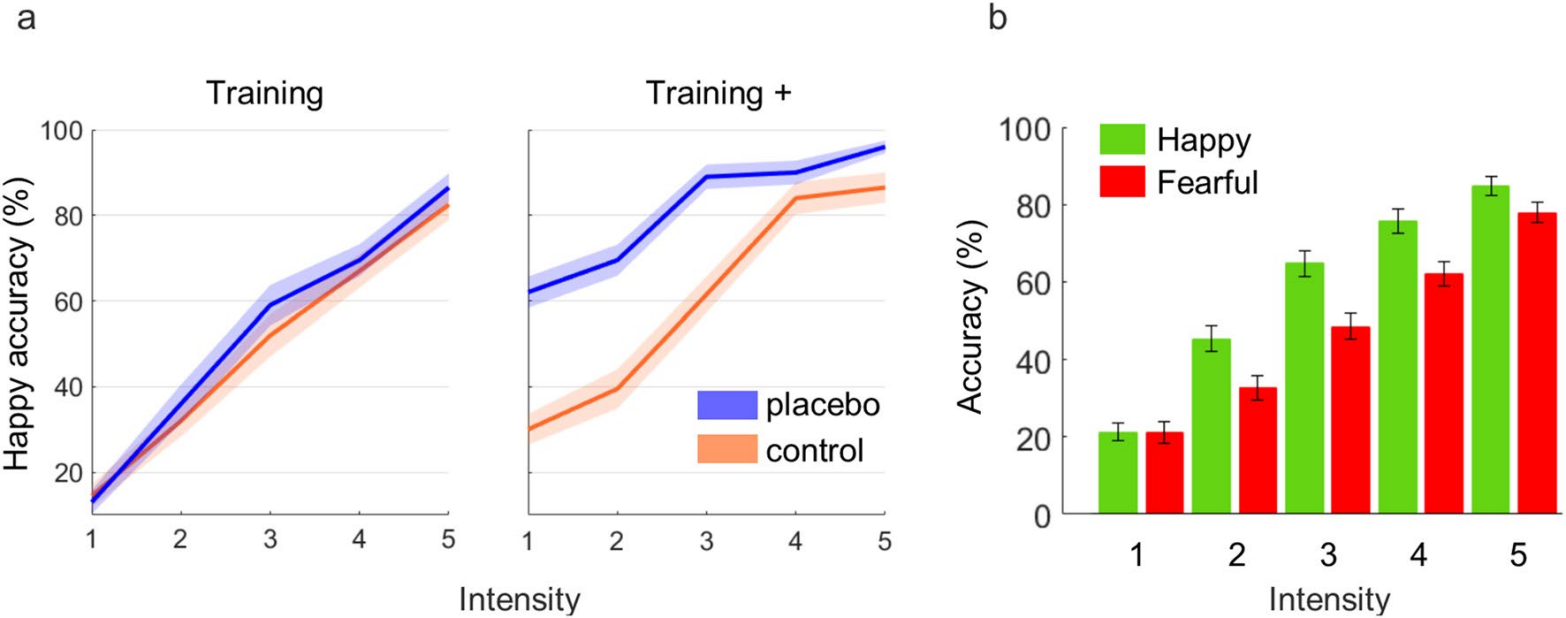
Classification accuracy. (**a**) Mean and s.e.m. of classification accuracy for happy expressions in training (left) and training + (right) sets for the placebo (blue) and control (orange) conditions. (**b**) Group means and s.e.m. of classification accuracy in the main task for happy (green) and fearful (red) expressions during the control condition at five steps of expression intensity.

**Figure 4:**
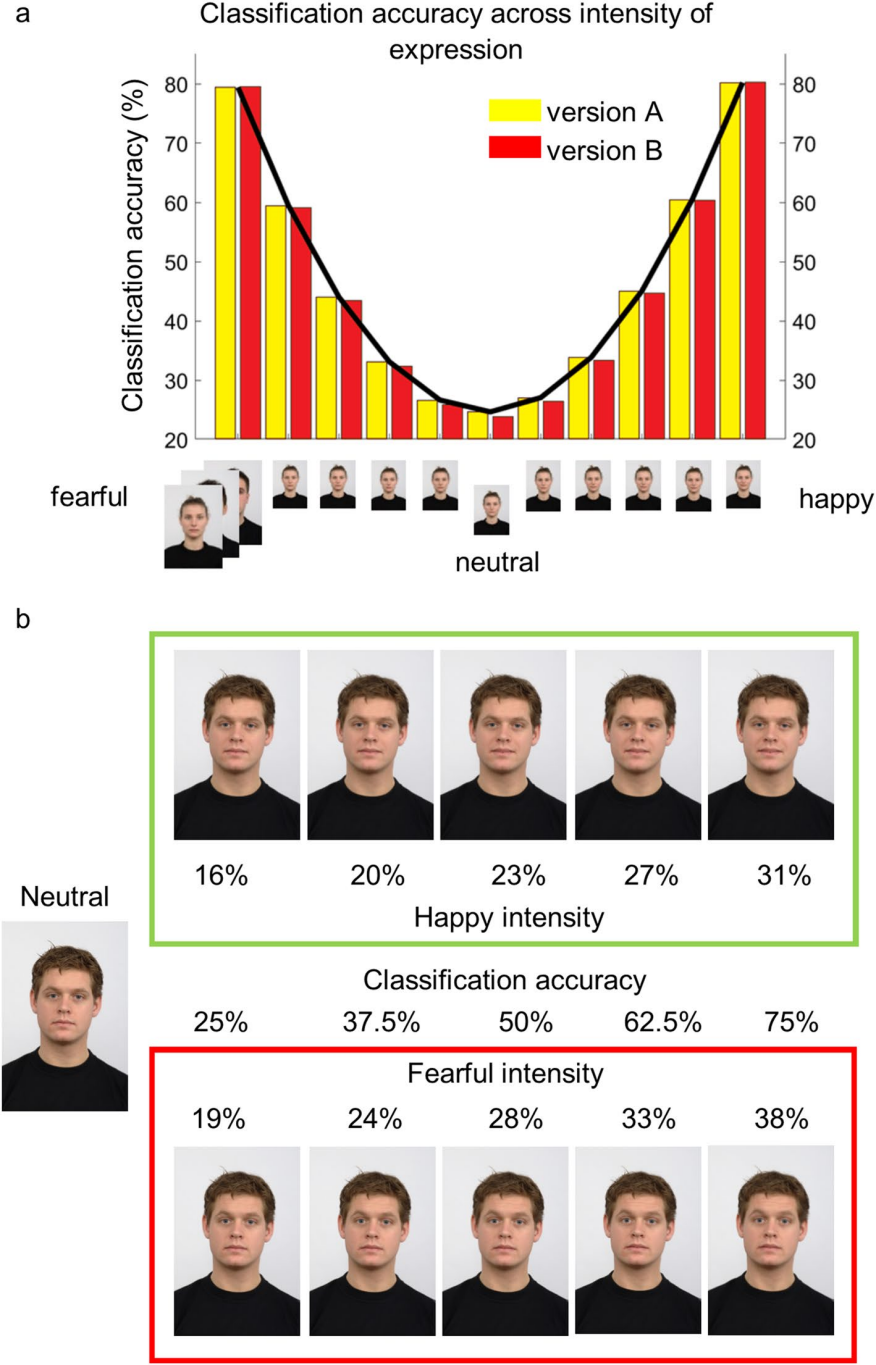
Matched stimulus sets and example stimuli. (**a**) Mean accuracy ratings of the two parallel stimulus sets as derived from the pilot study. (**b**) Example of stimuli used. Five steps of expression intensity for happy and fearful emotions and a neutral expression were used for each identity. Values presented in the colored boxes indicate the approximate expression intensity on the morphing dimension from 0% (neutral) to 100% (happy/fearful). Faces depicted are used with permission from the Radboud Face Database.

Pre and post nasal spray application, all participants performed a short training session of the emotion classification task whereby the post-treatment training at the placebo day used a manipulated stimulus set where the intensity of happy expression was slightly increased in order to simulate positive treatment effects. Validation of this covert manipulation was confirmed by a pilot study (N = 13), showing a specifically increased accuracy for happy expressions by this training set (t(12) = 4.41, p < 0.001, see “Methods” section) while 92% of participants were not aware of this manipulation. Replicating this validation data, in the current study this manipulation led to a specific improvement in classification accuracy for happy trials compared to the non-manipulated control condition (t(39) = 4.74, p < 0.001, Fig. 3a). No significant change in the classification performance of fearful expressions was observed (t(39) = 1.55, p = 0.128). Following these training sessions, all participants performed one of the two versions of the emotion classification task (Fig. 4a). At the control session, mean accuracy rates were 58% (± 16.6) for happy and 49% (± 17.4) for fearful faces (Fig. 3b). Ninety-one percent (± 11.2) of neutral faces were correctly classified. Thus, mean accuracy rates of around 50% for emotional faces nicely fit with our pilot data (see “Methods” section). Please note, that the slightly higher accuracy for happy faces does not reflect a confounding factor in this study since we were interested in within-subject contrasts.

**Figure 5:**
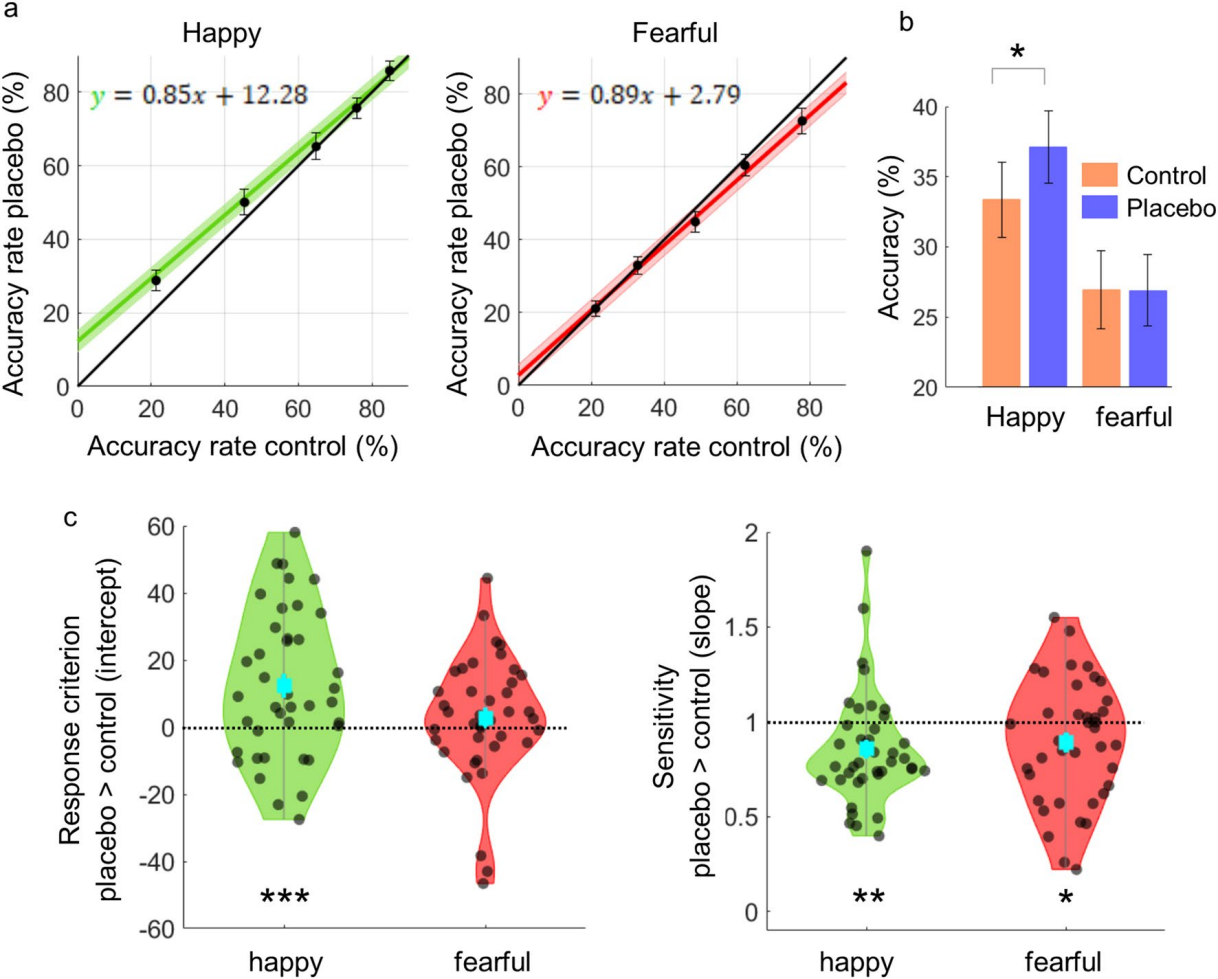
Response criterion and sensitivity in the emotional classification task. (**a**) Psychometric functions: Performance during the placebo condition was regressed onto performance during the control condition (s.e.m. of intercept shown as shaded area, error bars show s.e.m of condition differences at each step of expression intensity). (**b**) Condition differences for lowest 2 steps of expression intensity. Accuracy for happy expressions was greater in the placebo (blue) condition relative to the control (orange) condition specifically at the lower end of the intensity spectrum. No such differences were observed for fearful expressions. Error bars show s.e.m. (c) Distribution of slope and intercept values: The mean intercept for happy faces was significantly greater than zero, indicating a placebo-induced reduced threshold to detect happy faces. Mean slopes values for both emotions were significantly less than one, indicating a reduced ability to discriminate subtle emotional expressions under placebo. Mean and s.e.m are shown in the center of each violin plot. ***p < .001, **p < .005, *p < .05.

To investigate the effects of the placebo induction on emotion classification performance, we then regressed the individual data of the placebo condition onto the accuracy rates from the control condition. We thereby obtained a psychometric response function that expressed both an intercept and a slope value for each emotional expression, reflecting potential changes in response criterion and perceptual sensitivity, respectively (Fig. 5a). For happy facial expressions, results revealed the intercept values to be significantly greater than zero [M = 12.28, SD = 21.78] (t(39) = 3.56, p < 0.001, Fig. 5c). That is, participants used a more liberal response criterion for happy expressions in the placebo session. For fearful expressions, intercept values were not found to be different from zero [M = 2.79, SD = 18.20] (Fig. 5c). Thus, the tendency to label subtle emotional faces as fearful did not differ between the control and placebo condition (t(39) = 0.338 p = 0.338).

Further exploration of the data revealed that accuracy rates for happy faces were particularly improved under placebo for facial stimuli with only subtle emotional expressions (intensity of 16% and 20%, respectively) and accuracy rates below 50% (t(39) = 2.05, p = 0.046, Fig. 5b). We next assessed whether these changes are due to a general response bias towards positivity in the context of high uncertainty or whether participants actually improved their detection ability as would be reflected by an increased discrimination ability. To this end, emotion-specific slope-values of psychometric functions were tested against one, which would indicate no difference in discriminatory ability between the placebo and control session. Results revealed that for both happy [M = 0.85, SD = 0.85] and fearful [M = 0.89, SD = 0.31] faces, the slope values were significantly lower than one (t(39) = −3.07, p = 0.004 and t(39) = −2.13, p = 0.040, respectively). These findings indicate that positive expectation made participants less sensitive for differences in emotional expression in general but more likely to identify happiness even when only slight signs of happy expression were visible.

### Placebo effects on emotion detection are directly correlated with participants’ expectation and experience of mood enhancement

**Figure 6:**
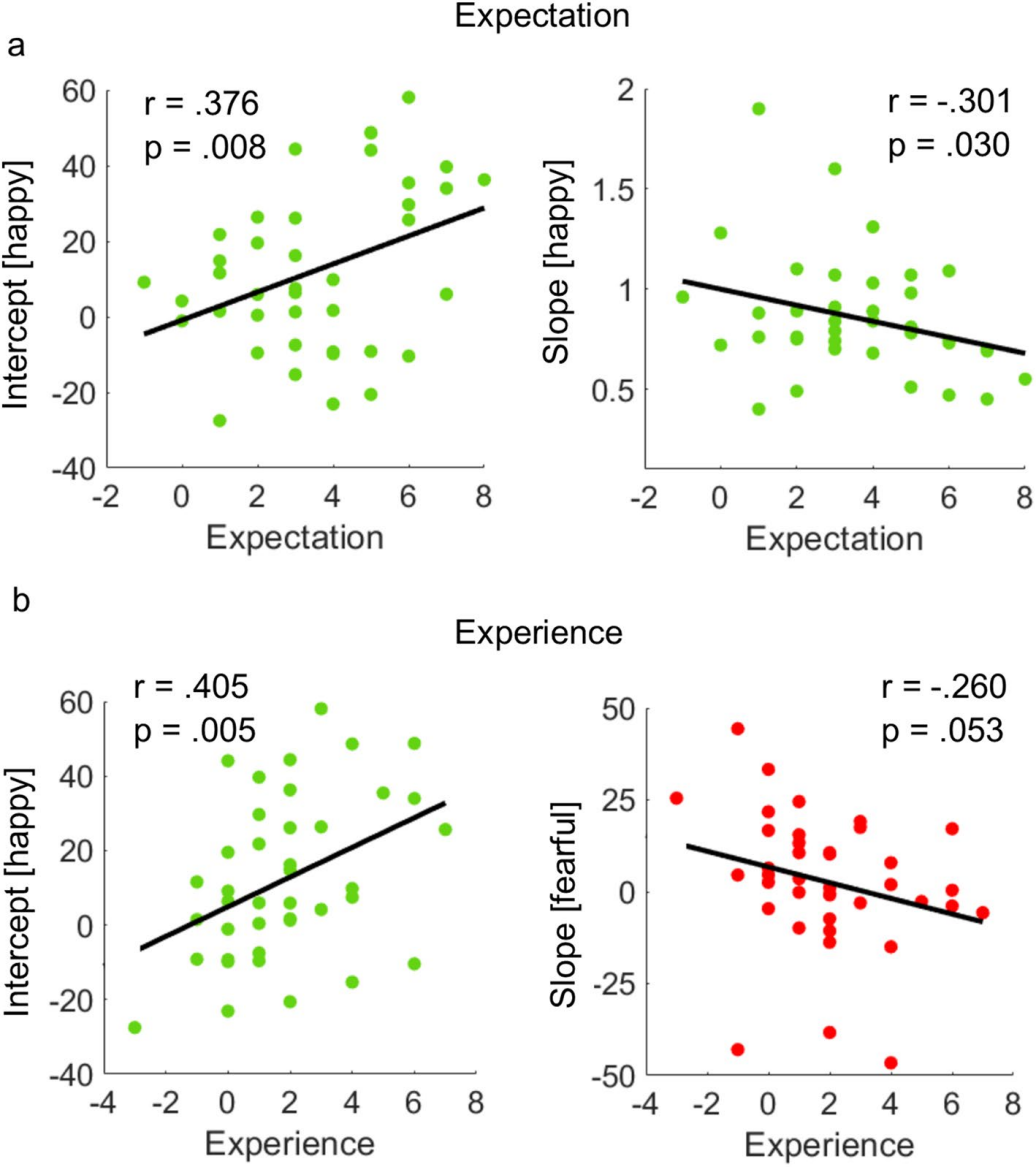
Correlations between self-reports and emotion classification measures. (**a**) Participants who reported a higher positive mood expectation, presented a more liberal response criterion (left) and reduced sensitivity (right) for happy expressions. (**b**) Participants who reported a higher positive mood experience presented a more liberal response criterion for happy expressions (left), and tended to apply a more strict response criterion for fearful expressions (right).

We next tested for direct relationships between these task-related changes and participants’ expectation of treatment effects. Results revealed that participants who reported stronger positive expectations following “oxytocin” application showed a stronger positivity effect in the placebo condition. Specifically, there was a positive correlation of reported expectations with placebo effects on intercepts (r(40) = 0.376, p = 0.008, Fig. 6a) and a negative correlation with placebo effects on slopes for happy faces (r(40) = −0.293, p = 0.033, Fig. 6a).

It could be assumed that task-related placebo-effects impact on participants’ retrospective experience of treatment effects. To test this, we correlated placebo-effects in our paradigm with reported positive treatment experience as assessed at the end of the study day. Here, we found participants who reported more positive experience to have demonstrated stronger placebo effects in the emotion classification paradigm. That is, positive experience of mood effects were correlated with increased intercept values for happy faces (r(40) = 0.405, p = 0.005, Fig. 6b). No such correlation was found using intercept values for fearful faces (r(40 = −0.260, p = 0.106) and consequently, both correlations differed significantly (Fisher’s z = 2.81, p = 0.002).

### Placebo effects on mood state were pronounced in treatment “believers”

Sixty-six percent of participants were convinced that they had received oxytocin on the placebo day. Using two-sample t-Tests, we assessed whether believers and non-believers differed in the aforementioned placebo effects. For expectation ratings and emotion classification measures, no such differences were observed (all p > 0.264). For experience ratings, believers reported stronger experiences relative to non-believers (t(38) = 2.93, p = 0.006) under placebo. This was also the case for VAS mood ratings. Believers reported better mood states than non-believers at the first (t(37) = 2.70, p = 0.010) and the second time point (t(37) = 2.90, p = 0.006). No participant believed to have received oxytocin when the instruction indicated the application of a saline solution.

## Discussion

This study set out to investigate whether the induction of positive expectation modulates emotional face perception in healthy individuals. Using an experimental protocol inspired by studies on placebo analgesia^27,29,32,34^, we could indeed demonstrate that expectations of positive treatment effects of an alleged intranasal oxytocin application lowered the overall threshold for identifying happiness in facial stimuli, especially when only subtle facial cues were present. At the same time, expression intensities were less well differentiated across emotions indicating this positivity effect to be highly specific. Individual placebo responses were directly correlated with treatment expectations and self-reported treatment effects. Moreover, placebo effects were accompanied by mood state improvement underlining the potential clinical relevance of positive expectations in the context of affective treatments.

The current findings are highly relevant to understand how expectations shape psychological processes such as face perception. For example, it has recently been discussed whether expectations can actually improve cognitive functions^31,32^. On the one hand, placebo-induced expectations have been shown to increase the subjective belief of behavioral improvements in the absence of actual changes in performance^32^. On the other hand, there are a few studies demonstrating expectation effects on reaction times and memory^31,35^. To the best of our knowledge, the current study is the first to explicitly address a potential enhancement of emotion recognition abilities by expectations. Importantly, our design allowed us to analyze psychometric response functions and thus to disentangle potential expectation effects on response tendencies (intercept) and on discriminatory sensitivity (slope). While our findings demonstrate a strong response bias towards the classification of happy faces, we also found participants to demonstrate a decreased ability to discriminate different intensities of emotional expressions when expecting the effects of oxytocin. Thus, individuals’ attention became less captured by subtle differences in the intensity of expressions and more focused on detecting happiness in relatively ambiguous stimuli. Neuroimaging findings on placebo-induced increased pleasantness of touch demonstrated such effects to rely on up-regulated prefrontal networks that were recruited through positive expectations^19^. With respect to the current study, one could speculate that placebo-induced expectations increased early sensory processing in face-sensitive brain regions such as the fusiform face area in response to even subtle facial cues which might have lowered the threshold for identifying happiness.

Most existing literature on positivity effects in facial emotion processing are from the field of healthy aging^21,22,34^. A selective focus on positive information such as happy faces^23,37^ has thereby been attributed to a motivational shift in goal-setting behavior^38^ and has been associated with better emotional well-being and attentional control^23,24,36^. The importance of top-down control has been further underlined by neuroimaging findings showing a critical role of ventromedial prefrontal and limbic networks in the generation of a positivity effect^23,39,40^. A similar emotion appraisal network is involved in mediating expectation effects in placebo analgesia^3,27^, hyperhedonia^19^ and the processing of affective stimuli^22^. The current finding of a selective bias in the classification of happy faces suggests a similar recruitment of top-down control by the expectation of oxytocin effects.

In patients with major depressive disorder, positive mood expectations and improvements have been shown^30,41,42^ and the extent of expectation is a significant mediator for the effectiveness of antidepressant treatment^30,43^. Importantly, we also observed a placebo-induced improvement of mood states in addition to the effects on face classification performance. Placebo effects on mood states in healthy individuals have been recently reported in some studies^44–46^ while others were not able to demonstrate such effects^47,48^. Formal models such as the predictive coding framework^49^ consider expectations as prior information, which, in a Bayesian sense, are integrated with incoming sensory information (likelihood), to form a percept (posterior)^3,50^. In the context of predictive coding, affective disorders have been linked with bottom-up deficits in predictive processing and an increased precision of negative prior beliefs^51^. The attentional negativity effect often observed in depression could be one consequence of such negative prediction bias. It is manifested, for example, in the tendency to interpret neutral or subtle facial expressions as negative^14,16^ and to generally prioritize the processing of negative information^15^. Treating depression could therefore be conceptualized as equipping the brain with the resources to modify its internal model of the world^52^, that is changing the brain's relevant statistical structures to become “less pessimistic”^53^. Antidepressant treatment has been shown to decrease a negativity bias while improving mood state^17,18^. In our healthy sample, we could show that expectation alone is able to modulate an attentional focus towards positivity and to improve mood. Given reports that up to 70–80% of treatment effects in antidepressant treatments are due to placebo mechanisms^5–7^, our findings highlight the possibility that this might at least be partly driven by expectation effects induced by the pharmacological intervention.

At the end of the last study day, we asked participants whether they thought they had received oxytocin at all. Although we acknowledge that such belief may vary gradually between participants, 1/3 of our sample denied this question and thus did obviously not believe our cover story. Exploratively contrasting ‘believers’ and ‘non-believers’ revealed differences in mood experience and online mood ratings. Similar to the parametric relationship of expectation ratings and placebo effects, participants who believed to have received oxytocin, expressed a larger degree of mood improvement during the placebo condition than those that did not. Though belief was not found to impact the induced positivity bias, it is evident that maintaining the belief of receiving an active treatment was instrumental for the experience of a positive mood change. These findings are consistent with other studies showing that previous experiences of successful treatments are an important intrinsic determinant of placebo responses^54,55^. Here, mood experience ratings provided at the end of each testing day indicated that positive expectation together with placebo effects significantly increased the retrospective experience of treatment effects which in turn may modulate positive priors for future treatment effects.

Despite several strengths including the detailed assessment of expectation, experience and mood changes in a well-controlled cross-over design and the isolated measurement of changes in response criterion and perceptual sensitivity, we acknowledge that the presented work is not void of limitations. First, the present study used a newly developed design in which a positive expectation induction included an active reinforcement of expectation, as was previously common in the context of placebo effects in for example pain^33,34^ and respiration^56^. Expectation ratings and effects on mood and task performance suggest that this manipulation was successful. However, in such experimental designs, it is impossible to determine whether placebo effects are a manifestation of learning mechanisms such as classical conditioning, expectancies, or both^57,58^. To understand the relative contribution of verbal instruction and classical conditioning for placebo effects in the affective system, future studies should acquire expectation ratings before and after an active reinforcement procedure such as the one used in the present study, or directly compare placebo effects following only verbal instruction or an additional conditioning procedure. Second, individuals differ in the nature by which they perceive and interpret emotional information^59^ and it has also been questioned to what degree laboratory studies on isolated, static facial expressions generalize to real-life conditions^60^. Therefore, the use of individualized, dynamic and rich stimulus sets could allow for a more precise understanding of the impact of positive expectation on the processing of subtle emotional cues and could allow for an accurate modelling of behavior at the individual level. Finally, we observed that mood state ratings decreased significantly following the emotion classification task relative to the ratings provided immediately following the expectation induction. This reduction could result from habituation or fatigue effects, and/or could reflect the rather short-lasting effects of our positive expectation induction procedure. Future work should aim to refresh participants’ expectations and to strengthen/maintain placebo effects, e.g. by implementing repeated feedback procedures.

To sum up, the current study could demonstrate a placebo-induced positivity effect and an increase in mood state following a newly developed procedure of expectation induction. The ability to induce a positive shift in one’s motivation to recognize subtle emotional stimuli in the absence of any active treatment in a healthy sample is promising and has relevant clinical implications. As such, we feel that the current findings should encourage future work on individuals with mood disorders in order to further elucidate the influence of expectations on emotional processing and whether the addition of a positive expectation induction could improve outcomes for the treatment of affective disorders.

## Methods

### Participants

We recruited 44 young adults for this study. Inclusion criteria comprised no current intake of prescribed medication, no history of psychiatric or neurological disorders, and normal or corrected to normal vision. Participants were recruited through an online advertisement and received financial compensation (€50) for taking part. Participants gave informed consent and ethical approval was granted by the local ethics committee (Ethikkommission der Ärztekammer Hamburg). All methods were performed in accordance with the relevant guidelines and regulations. Four participants needed to be removed due to extreme response behavior (more than 2.5 standard deviations above or below the mean performance in the emotion classification task) resulting in a final sample size of 40 participants (21 women, mean age = 25.61 years, SD = 4.44 years, range = 19–34 years).

The present study was preregistered on the open science framework (https://doi.org/10.17605/OSF.IO/C3JP4). Please note that the original pre-registration included two further sub-studies (singleton and Posner task) that needed to be cancelled due to logistical constraints (Covid pandemic). This had no impact on the pre-registered hypotheses, design, and analyses of the present study.

### Procedure

After successful screening, participants attended the lab on two days, separated by approximately one week (Fig. 1a). Both testing days involved identical procedures, with the exception of the expectation induction, which is described in detail below. On each day, participants sat in a comfortable chair with their eyes positioned ~ 100 cm from the center of a 24’’ color LCD screen with a resolution of 1920 × 1080 pixels and a refresh rate of 60 Hz. After an initial training session of the emotion classification task, participants received the “treatment”, reported their expectation of treatment effects, performed another training session (manipulated at ‘oxytocin treatment’ day) and started the task (Fig. 1b). Participants also reported their mood state via a digital visual analogue scale (VAS) at three time points throughout the testing phase as well as—in the form of an overall experience score—at the end of each day. Following completion of testing day 2, participants were asked whether they believed that they had received oxytocin during the study, and if so, on which study day.

#### Expectation Induction

To induce the expectation of the effects of intranasal oxytocin, participants viewed a self-developed 5 min video documentary, in which an expert explained the basic mechanisms of the neuropeptide, and elaborated on how oxytocin can positively modulate mood as well as the perception of emotional stimuli such as emotional facial expressions. Alongside detailed textual information, this allowed for a highly standardized expectation induction. The video was shown on both testing days 1 and 2 and was followed by the self-administration of four puffs (two in each nostril) of a saline nasal spray. Participants received the oxytocin information on one day (placebo condition), and the information to receive a saline solution (control condition) on the other with a counterbalanced order across participants. Participants were informed that it was possible to receive the same substance on both days. This was done to justify the identical setup on both study days. The allocated condition was revealed on the opening of a sealed envelope immediately after the video presentation. Deblinding was justified by the avoidance of potential placebo effects. On the day when participants allegedly received oxytocin, a hidden behavioral manipulation was accomplished within the second training phase of the emotion classification task (described below). This was included to reinforce the belief that oxytocin improved the sensitivity to detect positive emotional expressions. This hidden manipulation was not done on the control day when participants were informed that the solution was saline.

#### Emotion classification task

The emotion classification task was implemented with MATLAB (Mathworks, US)^61^ and the Psychophysics Toolbox extension^62^. The task required participants to label static images of emotional facial expressions of varying intensity as either happy, fearful, or neutral. Participants were informed that some of the faces contained very subtle emotional cues. Stimuli were selected from the Radboud Faces Database (https://rafd.socsci.ru.nl/RaFD2/RaFD?p=main)^63^ and morphed using Abrosoft Fantamorph^©^, so as to generate a continuum of emotional facial expressions from neutral to happy, and from neutral to fearful. Two pilot studies were conducted in order to create the two matched stimulus sets needed in the experiment (Fig. 4a). In the first pilot study, ten participants rated 30 identities (15 female) at 23 steps of emotional expression intensity (from 8 to 50% intensity in 2% increments, and an additional 60% intensity expression) in both happy and fearful emotions, along with a neutral expression for each identity (each stimulus presented eight times, resulting in a total of 5640 trials per participant). Of these 30 identities, 21 were chosen for the main experiment based on the quality and shape of their induced response functions. Specifically, detection accuracy values derived from the pilot data allowed us to select images per emotion that produced 25%, 37.5%, 50%, 62.5%, and 75% of classification accuracy rates (Fig. 4b). This centered the intensity range on 50% of detection accuracy (i.e., the indifference point). Based on these specifications, we created two stimulus sets that were matched for difficulty both across sets, and across both emotions. For happy facial expressions, these steps reflected approximately 16%, 20%, 23%, 27%, and 31% of happy intensity. For fearful facial expressions, the 5 steps corresponded to approximately 19%, 24%, 28%, 33%, and 38% of fearful intensity.

**Figure 7:**
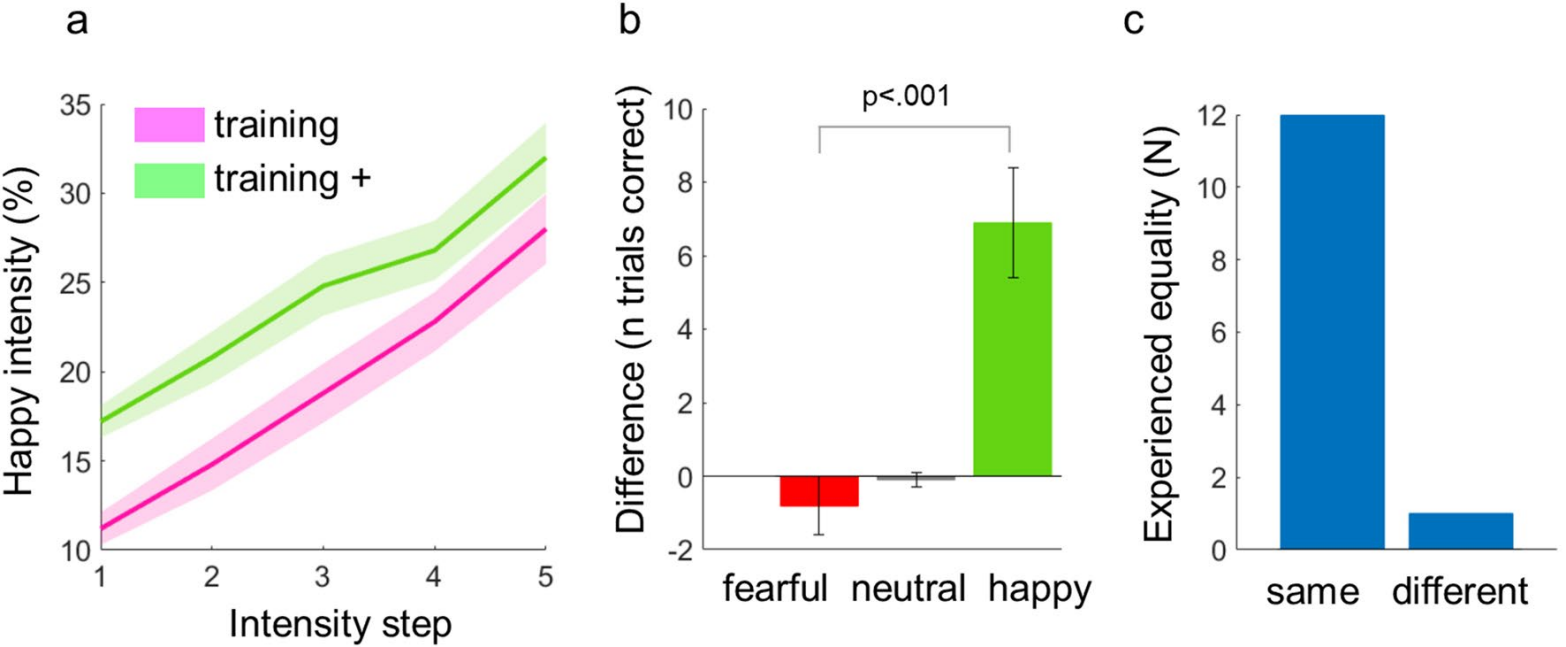
Pilot data for generating the manipulated training set. (**a**) Percentage of happy expression in the training and the manipulated training + sets. Shaded area shows s.e.m. (**b**) Mean accuracy difference between training and training + and s.e.m. Accuracy for happy expressions was significantly increased compared to fearful expressions. (**c**) Perceived equality of training and training + stimulus sets.

The final task began with a ‘training’ phase, whereby individuals had to label 50 images from 5 identities not included in the experimental sets, with varying intensity of happy and fearful facial expressions as well as 5 neutral faces. Participants then viewed the video documentary on oxytocin, and were informed whether they were to receive ‘oxytocin’ (placebo) or saline (control) on the current day. After the administration of the nasal spray, participants then performed a second training phase (‘training + ’), including 55 images from another 5 different identities. On the placebo day, the intensity of happy expressions was subtly increased in order to specifically facilitate the recognition of happy faces and thus to reinforce the belief in oxytocin effects. The effectiveness of this shift was determined by a second pilot study (N = 13). Pilot data showed that an increase of 4–6% in happy expression intensity (Fig. 7a) was sufficient for improving detection accuracy of happy faces by 16% (Fig. 7b), while maintaining the subjective belief that both training and training + sets were identical (Fig. 7c). On the control day, images in the first and second training phase were identical.

Following the second training, participants performed one of the two parallel versions of the main task (counterbalanced across testing days). Trial orders were pseudo-randomized in order to ensure no consecutive presentation of the same identity.

The paradigm consisted of 352 trials per set with 8 identities (4 female) showing the five intensities of happy and fearful expressions as well as the neutral expression (see Fig. 3b for an example). Each picture was presented four times. This resulted in 32 trials per intensity step, 160 trials per emotion, and an additional 32 neutral trials. A fixation cross was presented for 0.5 s, which was followed by the face stimulus for 1.5 s (horizontal = 7.72° and vertical = 11.57° of visual angle). Once the face disappeared, response options were presented, and participants were required to select ‘fearful’, ‘neutral’, or ‘happy’ by pressing the appropriate key on the keyboard. The response option that was selected was highlighted for 165 ms (Fig. 1b).

#### Mood ratings (visual analogue scale; VAS)

Mood ratings were collected at the beginning of the testing day (t0 = baseline), after training + (t1), and after completing the emotion classification task (t2). Participants were requested to move a randomly positioned cursor on a scale that ranged from ‘unhappy’ to ‘happy’ with a resolution of 200 steps, by using the left or right arrow keys on the keyboard.

#### Expectation and experience assessment

After the nasal spray application, participants reported on an 11 point scale the degree to which they expected a positive mood change (0: expected no positive mood change, 10: expected a large increase in mood). At the end of each study day, they reported their general experience of a positive mood change (0: experienced no positive mood change, 10: experienced a large positive mood change). Rating scales were adapted from Rief et al.^64^.

#### Belief of the administration of oxytocin

At the end of the second testing day, participants were asked whether they believed that they had received oxytocin during the study, and if so, at which study day.

### Data analyses

Data processing and statistics were conducted using MATLAB (Mathworks, MA) and SPSS 27 (IBM, NY). We report statistical tests from the general linear model framework, including one-sample t-tests, paired t-tests, repeated measures analysis of variance (rmANOVA), and Pearson correlations. Statistical significance was assumed based on an alpha value of 0.05.

#### Power analysis

Previous data on positive expectation effects using the same manipulation as in the present study report large effect sizes (d = 1.06;^17^). To increase sensitivity, we powered the present study to detect medium-effect sizes (d = 0.50) of the expectation manipulation, assuming an alpha of 5% and a power of 90%. This resulted in a required sample size of N = 44. Post-hoc sensitivity analysis for our final sample size (N = 40) results in a slightly increased effect size threshold of d = 0.52.

#### Analysis of mood state

For each participant, VAS values were scaled relative to the minimum and maximum values provided on a given testing day in order to account for individual differences in the range of the scale used. Values were baseline corrected by subtracting t0 from t1 and t2 values, respectively.

#### Emotion classification task

Accuracy values were calculated for both placebo and control conditions as well as happy and fearful expressions separately. This was achieved by deriving the proportion of trials correctly labelled as showing the respective emotional expression within each intensity step relative to the number of trials presented for a given step. Detection performance at each step of expression intensity in the control condition was considered as baseline. Performance at each intensity step for the placebo condition was regressed onto the control condition performance within each participant, allowing us to derive a linear regression function for each emotional expression that provided an intercept and a slope value, in order to directly evaluate changes in response criterion and sensitivity in the placebo compared to the control condition.

##### Measurement of response criterion

The intercept value of the derived linear function was used to quantify the response criterion for each emotion. An intercept of zero would indicate that the response criterion did not change as a function of placebo administration. If the intercept value was found to be greater or less than zero for a specific emotion, this would indicate a placebo-induced shift in the response criterion, i.e. participants use an increased or decreased threshold to label a subtle emotional facial expressions as belonging to a specific emotional category. A value greater than zero would indicate a more liberal response criterion, whereas a value less than zero would indicate a stricter criterion.

##### Measurement of discrimination ability

The slope value provides a measure of the ability to discriminate subtle emotional expressions^65^. The steepness of the slope represents the extent of the increase or decrease in the percentage of detection sensitivity per unit change on the x-axis (here, detection performance per step relative to the control condition). A slope value of one would indicate that the sensitivity to discriminate subtle expressions did not change as a function of placebo (i.e., performance was equal for both conditions). If the sensitivity was to increase under placebo, then the slope would become steeper and would assume a value greater than one, indicating that small changes in emotion intensity yield larger changes in the perception of the respective emotional expression. By contrast, a slope value less than one would indicate that the discrimination sensitivity was reduced for the placebo condition relative to the control condition.

## Data Availability

The datasets generated and/or analyzed during the current study are available in the Research Box repository (https://researchbox.org/598).
